# Infection by a eukaryotic gut parasite in wild *Daphnia* sp. associates with a distinct bacterial community

**DOI:** 10.1093/femsec/fiac097

**Published:** 2022-08-26

**Authors:** Amruta Rajarajan, Justyna Wolinska, Jean-Claude Walser, Minea Mäder, Piet Spaak

**Affiliations:** Department of Aquatic Ecology, Swiss Federal Institute of Aquatic Science and Technology (Eawag), 8600 Dübendorf, Zürich, Switzerland; Department of Evolutionary and Integrative Ecology, Leibniz Institute of Freshwater Ecology and Inland Fisheries (IGB), 12587 Berlin, Germany; Department of Biology, Chemistry, Pharmacy, Institut für Biologie, Freie Universität Berlin (FU), 14195 Berlin, Germany; Department of Environmental systems science (D-USYS), Genetic Diversity Centre (GDC), Federal Institute of Technology (ETH) Zürich, 8092, Zürich, Switzerland; Department of Aquatic Ecology, Swiss Federal Institute of Aquatic Science and Technology (Eawag), 8600 Dübendorf, Zürich, Switzerland; Department of Aquatic Ecology, Swiss Federal Institute of Aquatic Science and Technology (Eawag), 8600 Dübendorf, Zürich, Switzerland

**Keywords:** 16S rDNA, *Caullerya*, disease ecology, infection, microbiome, zooplankton

## Abstract

Host-associated bacterial communities play an important role in host fitness and resistance to diseases. Yet, few studies have investigated tripartite interaction between a host, parasite and host-associated bacterial communities in natural settings. Here, we use 16S rRNA gene amplicon sequencing to compare gut- and body- bacterial communities of wild water fleas belonging to the *Daphnia longispina* complex, between uninfected hosts and those infected with the common and virulent eukaryotic gut parasite *Caullerya mesnili* (Family: Ichthyosporea). We report community-level changes in host-associated bacteria with the presence of the parasite infection; namely decreased alpha diversity and increased beta diversity at the site of infection, i.e. host gut (but not host body). We also report decreased abundance of bacterial taxa proposed elsewhere to be beneficial for the host, and an appearance of taxa specifically associated with infected hosts. Our study highlights the host-microbiota-infection link in a natural system and raises questions about the role of host-associated microbiota in natural disease epidemics as well as the functional roles of bacteria specifically associated with infected hosts.

## Teaser

Water fleas infected with a gut parasite have a distinct microbiome in their guts and bodies.

## Introduction

Host-associated bacterial communities play an important role in host health and the outcome of infectious disease. Several studies document host-associated bacteria's role in increased colonization resistance against parasites (Libertucci and Young 2019, Sekirov *et al*. 2010). Host-associated microbiota may be beneficial to the host by contributing to the activation of the host's adaptive immune response to the pathogen and/or competitively exclude pathogens by monopolizing host resources (Buffie and Pamer 2013, Sorbara and Pamer 2019).

However, bacterial community members that are beneficial or commensal to the host may also indirectly contribute to increased susceptibility to infectious disease. For instance, commensal members of the host-associated bacterial community that are generally present in the host in the absence of a pathogenic infection could expand into newly developed niches within the host upon disruption by a pathogen, hence switching to a pathogenic lifestyle (Bliska *et al*. 2021). The bacterial pathogen *Bacillus thuringiensis* is lethal to its spongy moth host in the presence of a host-associated bacterial community, but not in antibiotic-treated hosts (Broderick *et al*. 2006) and similarly, germ-free hosts infected with the fungal pathogen *Beauveria bassiana* survive longer than infected hosts with microbiota (Lai *et al*. 2017). Thus, host-associated microbiota may either increase or decrease host susceptibility to infection by a pathogen. In general, a diseased state in the host may result from complex interactions between the host, associated microbes and the pathogen (Bass *et al*. 2019, Bernardo-Cravo *et al*. 2020, Pitlik and Koren 2017).

There is now an increased appreciation for the putative role of bacterial communities in natural epidemics of infectious diseases (Stencel 2021). Infection by a parasite leads to disease-induced dysbiosis or progressive changes in the host's bacterial community structure, with late-stage infections typically correlating with a lower diversity of associated microbes (Chen *et al*. 2017, Jani and Briggs 2014, Lloyd and Pespeni 2018, Nunez-Pons *et al*. 2018, Rosado *et al*. 2019) and an increased occurrence of opportunistic pathogens (Cornejo-Granados *et al*. 2017, Griffiths *et al*. 2019). An increase in dysbiosis-related bacteria in a host caused by a pathogenic infection (or an ecological stressor) may even increase susceptibility of a host to disease (Boutin *et al*. 2013, Gustin *et al*. 2021, Hinderfeld and Simoes-Barbosa 2020). Hence, an understanding of host-associated microbial community changes in response to infectious disease epidemics has implications in wildlife disease management in both terrestrial (Allender *et al*. 2018, Denman *et al*. 2018, Woodhams *et al*. 2014) and aquatic (Luter *et al*. 2017, Meyer *et al*. 2019, Quintanilla *et al*. 2018, Vezzulli *et al*. 2013) systems.

Conversely, commensal host-associated bacterial communities may play a role in susceptibility to infections and consequent epidemics—though studies investigating this have been limited due to the challenging nature of inferring causal relations in the field. Soil microbiota dynamics influence the development of protistan clubroot disease in the Rutabega plant, *Brassica napus* (Daval *et al*. 2020). Populations of the common frog *Rana temporaria* that experienced repeated epidemics of *Ranavirus* had distinct bacterial communities compared to populations that did not (Campbell *et al*. 2019). Similarly, populations of the common Midwife toad that experience large, recurrent epidemics of the fungal pathogen *Batrachochytrium dendrobatidis* are associated with a distinct skin microbiota, which is not explained by distinct pathogen genotypes across sites (Bates *et al*. 2018). Further, epidemiological dynamics of *B. dendrobatidis* are linked with host-associated bacterial communities (Jani *et al*. 2017, Longo *et al*. 2015).

The zooplankter water flea *Daphnia* is a dominant species in freshwater ecosystems (Lampert 2011). It reproduces by cyclical parthenogenesis, with clonal reproduction being the dominant reproductive mode, and sexual reproduction occurring only under unfavourable environmental conditions. Two studies have investigated the role of *Daphnia-*associated bacterial communities during a parasite infection. Gut bacteria were found to play no role in *Daphnia magna* susceptibility to its bacterial pathogen *Pasteuria ramosa* (Sison-Mangus *et al*. 2018). Also, germ-free *D. magna* that received microbiomes of *Daphnia* pre-exposed to a mixture of parasites did not show increased survival or tolerance upon re-exposure to the same parasites (Bulteel *et al*. 2021). Both studies investigated the influence of host-associated bacterial communities on *Daphnia* infection patterns under laboratory settings. However, no studies so far have investigated links between host-associated bacterial communities and parasite infections in wild *Daphnia* populations.

Populations of the *Daphnia longispina* complex in the lake Greifensee are infected with the highly virulent, eukaryotic gut parasite, *Caullerya mesnili* (Gonzalez-Tortuero *et al*. 2016, Wolinska *et al*. 2004) (Family: Ichthyosporea) (Lu *et al*. 2020). Epidemics of *C. mesnili* (hereafter referred to as *Caullerya*) are seasonal and peak during late autumn and winter (Turko *et al*. 2018). Parasitization by *Caullerya* exerts a strong selection pressure on the host (Schoebel *et al*. 2010, Turko *et al*. 2018) by drastically reducing host fecundity and increasing mortality (Lohr *et al*. 2010). In this study, we investigate the relationship between infection by *Caullerya* and host-associated bacterial community composition in the *D. longispina* complex during a natural epidemic of the parasite. Specifically, we identify bacterial taxa that may uniquely associate with *Caullerya-*infected *Daphnia*. For this, we profiled bacterial communities of infected and uninfected hosts (in both their guts and body tissue) using 16S rRNA gene amplicon sequencing. We hypothesized that: (i) alpha diversity of gut bacterial communities would be lower in infected compared to uninfected hosts, consistent with pathogen-mediated dysbiosis, (ii) beta diversity of gut bacterial communities should differ between infected and uninfected hosts, reflecting colonization by putatively opportunistic bacterial pathogens and/or dominance of bacterial taxa related to dysbiosis in infected hosts, and (iii) differences in alpha and beta diversity of bacterial communities between infected and uninfected hosts should be limited to gut tissue (i.e. host body tissue should not differ in their bacterial communities by infection status) since the infection is specific to the gut.

## Material and methods

### *Daphnia* sampling and processing

All *Daphnia* in the study were collected on 22/12/2020 from lake Greifensee (N 47°20′41″, E 8°40′21″) from a single sampling location using four vertical tows (0–30 m) with a 150 µm mesh plankton net (Turko *et al*. 2018). The zooplankton sample was transferred to a 10 L canister half-filled with surface lake water from the same location. In this study, we define a *Caullerya* ‘infection’ as visible, late-stage infection. A late stage *Caullerya* infection presents as spore clusters in the *Daphnia* gut, typically 8–12 days after initial exposure to the parasite (Lohr *et al*. 2010). For identifying *Caullerya-*infected *Daphnia*, zooplankton in the sample were collected on a ∼500 µm mesh and *Daphnia* were visually screened for the presence of *Caullerya* spores in the gut under 40–50x magnification (Lohr *et al*. 2010). Ten adult *Daphnia* (>1 mm in size) containing *Caullerya* spores in the gut were gently lifted by the antennae using sterilized forceps and placed by either PS or MM in a bottle containing 10 ml lake water filtered through a 0.45 µm mesh. For uninfected *Daphnia*, 10 adult *Daphnia* that did not have visible *Caullerya* spore clusters in the gut were collected in the same way. No criteria other than the size cut-off, exclusion of individuals carrying resting eggs (indicating sexual reproduction) and presence/absence of *Caullerya* spores were used to exclude *Daphnia* from the study. Animals were not screened for the presence of other infections; however, infections other than *Caullerya* are rare in this *Daphnia* population, especially in winter (Tellenbach *et al*. 2007, Wolinska *et al*. 2004). Forceps were wiped with 10% bleach between every animal picked up to minimise cross-contamination of spores and/or bacteria. Sixteen bottles each of 10 infected and 16 bottles each of 10 uninfected *Daphnia* (total of 160 infected and 160 uninfected *Daphnia*) were prepared in alternating order. This was done to ensure that infected and uninfected *Daphnia* were processed from collection to dissection at the same average duration. These animals were later dissected live under a stereomicroscope (see below) with either gut or body tissue of 20 animals pooled into 1.5 ml microcentrifuge tubes per replicate (see ‘Pre-processing of reads’ for final sample size after sequencing).

### Dissection and sample preparation

Sets of *Daphnia* picked into 10 ml bottles (see above) were used for the preparations of gut bacterial and body bacterial community samples. For gut samples, 20 *Daphnia* per replicate were dissected under a stereo microscope, each in individual droplets of nuclease-free water using sterilized forceps (these 20 *Daphnia* originated from two bottles of 10 *Daphnia*). Extracted guts were immediately pooled into a separate 20 µl droplet of nuclease-free water, and afterwards transferred with a pipette to a 1.5 ml microcentrifuge tube containing 10 µl nuclease-free water. For paired body samples, the remaining tissue after extraction of guts was similarly pooled into a separate 1.5 ml microcentrifuge tube. Forceps were wiped with 10% bleach between every dissection to minimize cross-contamination between *Daphnia* gut and body samples. All *Daphnia* were dissected within 24 h of being collected from the lake. All samples were stored at −20°C immediately upon dissection until further processing. Four dissection negative controls (each containing 30 µl of the water used to dissect *Daphnia*) were also prepared.

### Bacterial community profiling

DNA was extracted using the Qiagen Blood & Tissue kit (Cat #69506). Two DNA extraction blanks (tubes without biological material) were added to the workflow. All samples were lysed at 56 °C for four hours and the extraction protocol provided by the manufacturer was followed. Samples were eluted in 40 µl kit elution buffer for 20 min. A nested PCR approach was used to account for low biomass of our samples; at this stage, two PCR no-template controls were added to the workflow. Barcoded universal 16S rRNA gene primers 515F (5′-GTGCCAGCMGCCGCGGTAA-3′) (Caporaso *et al*. 2011) and m806R (5′-GGACTACNVGGGTWTCTAAT-3′) (Apprill *et al*. 2015) were used to amplify the V4–V5 region of the 16S rRNA gene using the following cycling conditions: initial denaturation at 95°C–3 min; (98°C–20 s, 52°C–15 s, 72°C–15 s) 32 cycles; followed by a final extension at 72°C for 5 min. This PCR step was done in triplicate for each sample, and triplicates were pooled before PCR purification. The location of all samples (including a total of eight negative controls) in the 96-well plates was randomized, though triplicates were always adjacent. PCR products were then purified using magnetic Agencourt AMPure XP beads (Cat #A63881) before being used in a second PCR with indexing primers. Indexes from the Illumina Nextera XT V2 Library Prep Kit were added to the samples via a PCR, under the conditions: initial denaturation at 95°C–3 min; (95°C–30 s, 55°C–30 s, 72°C–30 s) 9 cycles; followed by a final extension at 72°C for 5 min. Indexed PCR products were purified again using AMPure beads. The samples were then quantified using a Qubit HS Assay, normalized and pooled into a 1.5 nM library for amplicon sequencing. Paired-end sequencing was carried out using MiSeq–600 cycles (PE300) v3 run kit with 10% PhiX.

### Pre-processing of reads

Sequencing resulted in a total of 11.6 M paired-end reads (minimum = 204312, maximum = 519591 per biological sample). After exclusion of samples that failed sequencing (<200 reads), the following number of biological replicates remained in the study: 8 samples of infected guts, 7 of infected bodies, 7 of uninfected guts and 7 of uninfected bodies, representing a total of 160 *Caullerya-*infected and 140 uninfected individuals. Initial pre-processing steps were performed on the Euler computing cluster at ETH Zürich. Raw reads were 5′-end trimmed, merged, and quality filtered. Amplicons were clustered using UPARSE2 (Edgar 2013), denoised into zero-radius Operational Taxonomic Units (ZOTUs) using UNOISE3 (Edgar 2016), further clustered based on 97% sequence similarity and annotated using non-Bayesian SINTAX classifier v.138 (Edgar 2016) and the Silva (v138) database (Quast *et al*. 2013). All subsequent steps were performed in R v4.0.2 using the package phyloseq (McMurdie and Holmes 2013). 29 ZOTUs (14 of unidentified phyla, 13 chloroplasts, 2 mitochondria) were filtered from the dataset, resulting in a total of 737 ZOTUs. ZOTUs discovered in negative controls were not removed, as this was not suitable for our dataset; see Fig S1 and Table S1 for a detailed description of negative controls and their analyses and also (Hornung *et al*. 2019, Kim *et al*. 2017). All samples were then rarefied to an even depth of 170 000 reads. The SRS method of normalizing library sizes (Beule and Karlovsky 2020) yielded the same statistical results as rarefaction and hence only the results of the rarefied dataset are presented (see below). The rarefaction step resulted in the removal of 54 ZOTUs, leaving 683 ZOTUs in the dataset.

### Statistical analyses

All statistical analyses were done in R v4.0.2 using the phyloseq package. First, diversity indices were compared between *Caullerya-*infected and uninfected *Daphnia*, separately for gut and body tissue since we were primarily interested in the effects of infection. All diversity analyses were performed at the ZOTU level. Alpha-diversity measures (qualitative: ZOTU richness and quantitative: Inverse Simpson Index) of samples were estimated using the *estimate_richness* function. One-way ANOVAs were performed on both alpha diversity measures to test for variation between infected and uninfected *Daphnia*. Next, beta diversity indices (qualitative: Jaccard dissimilarity and quantitative: weighted unifrac distance) were visualised using PCOA plots. One-way PERMANOVAs were performed using the *adonis* function of the vegan package (9999 perm). PERMANOVAs of beta diversity metrics were only performed after checking if the data meets the homogeneity of dispersion assumption using the *betadisper* function of the vegan package. Percentage variation explained by the models used for beta diversity analyses was estimated with a db-RDA using the *capscale* function.

Second, to identify ZOTUs indicative of *Daphnia* infection status, the *signassoc* function of the Indicspecies package in R was used (two-tailed test, 9999 perm, corrected for multiple comparisons using the Sidak method) (De Caceres and Legendre 2009). This analysis identifies specific ZOTUs with skewed abundance distributions across groups. This was done on pooled gut and body tissue since we aimed to identify bacterial taxa that associate significantly with *Daphnia* infection status regardless of host tissue. The relative abundances of indicator ZOTUs were then visualised in a heatmap using ggplot2.

Finally, the dominant bacterial orders in *Daphnia* guts and bodies were visualised. ZOTUs were aggregated at the order level using the phyloseq:: *tax_glom* function. Rare orders were lumped into the category ‘Other’ (i.e. orders constituting <1% of biological samples and not present in every sample). Differential abundance of dominant bacterial orders was tested between *Caullerya-*infected and uninfected *Daphnia*, separately for gut and body bacterial communities using the Wald test of the DESeq2 package (Love *et al*. 2014) and corrected for multiple comparisons using the ‘fdr’ method.

## Results

### Alpha diversity

**Figure 1.**
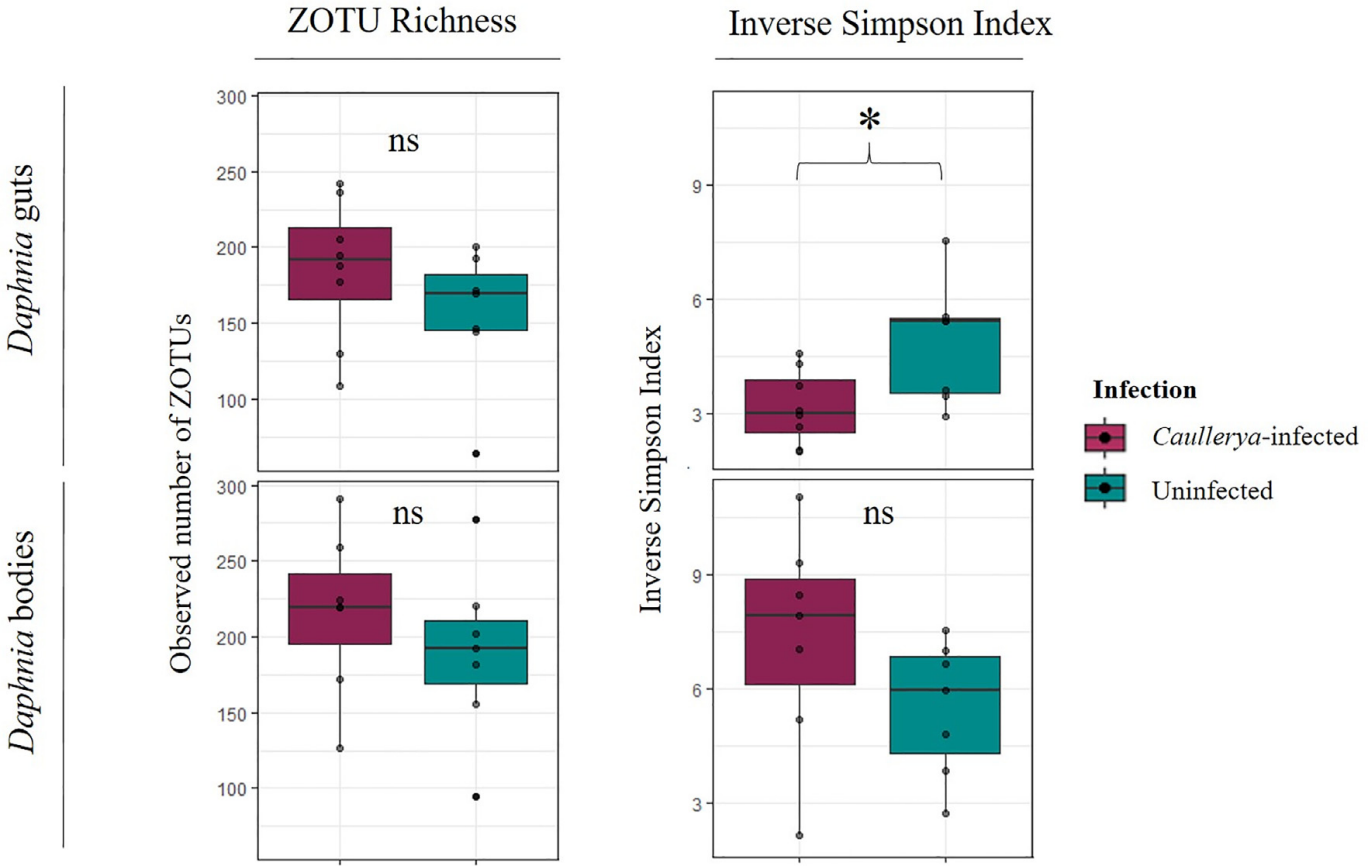
Alpha diversity indices: ZOTU richness (left panels) and Inverse Simpson Index (right panels) in *Caullerya*-infected (maroon) and uninfected (green) *Daphnia*-associated bacterial communities. Panels are indicated with tissue type; with *Daphnia* guts on top panels and *Daphnia* body in bottom panels, for each metric. *P < 0.05, one-way ANOVA.

We used ZOTU richness (qualitative) and Inverse Simpson Index (quantitative) measures of alpha diversity. ZOTU richness did not vary between *Caullerya-*infected and uninfected *Daphnia* regardless of host tissue (Fig. 1). The Inverse Simpson Index was significantly lower in *Caullerya-*infected guts compared to uninfected guts (F = 6.2, *P =* 0.027); however, *Daphnia* bodies showed an opposite (although not significant) trend (Table 1).

**Table 1.** One-way ANOVAs of alpha diversity metrics ZOTU richness (A) and Inverse Simpson Index (B) across *Caullerya*-infected and uninfected *Daphnia* separately for *Daphnia* gut and body samples. Model used was diversity metric ∼ infection. *P < 0.05

(A)	ZOTU Richness					
**gut**		Df	Sum Sq	Mean Sq	F value	*P* value
	infection	1	3320	3320	1.56	0.23
	Residuals	13	27 655	2127		
**body**		Df	Sum Sq	Mean Sq	F value	*P* value
	infection	1	2498	2498	0.83	0.38
	Residuals	12	36 270	3022		
**(B)**	**Inverse Simpson Index**					
**gut**		Df	Sum Sq	Mean Sq	F value	*P* value
	infection	1	10.5	10.46	6.2	0.027*
	Residuals	13	21.9	1.69		
**body**		Df	Sum Sq	Mean Sq	F value	*P* value
	infection	1	11.3	11.3	1.95	0.19
	Residuals	12	69.6	5.8		

### Beta diversity

**Figure 2.**
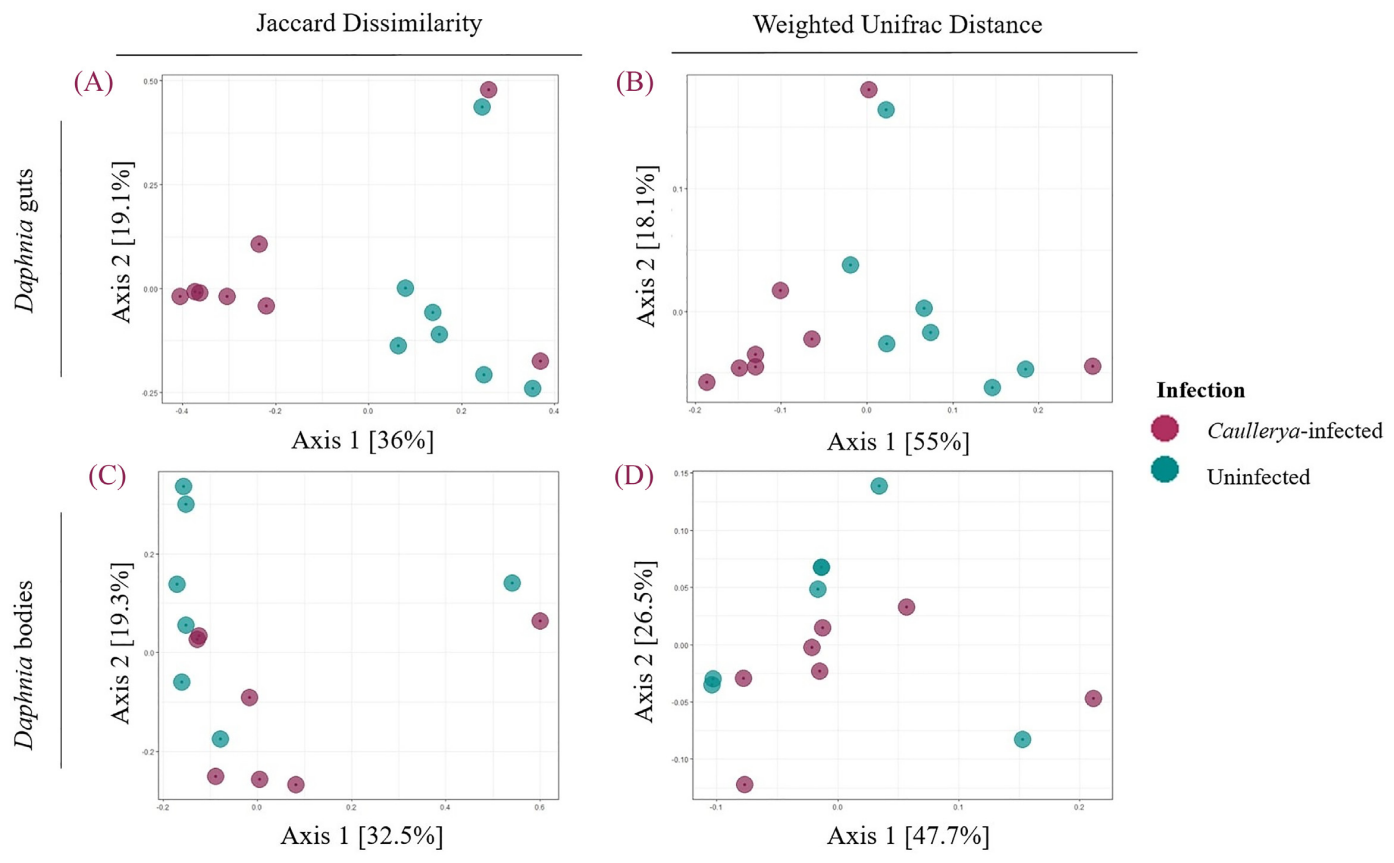
PCOA plots of beta diversity measures of *Daphnia* gut bacterial communities (top panels) and *Daphnia* body bacterial communities (bottom panels). Jaccard Dissimilarity (Fig. 2A and C) and Weighted Unifrac Distance (Fig. 2B and D); colors represent *Caullerya*-infected (maroon) and uninfected (green) bacterial communities.

We used the Jaccard Dissimilarity (qualitative) and Weighted UniFrac distance (quantitative) measures of beta diversity to compare bacterial community composition between infected and uninfected hosts. Jaccard Dissimilarity which is a count of the number of unshared taxa, differed significantly between *Caullerya-*infected and uninfected *Daphnia*, in both the guts (Fig. 2A, F = 3.01, *P* = 0.0093) and bodies (Fig. 2B, F = 2.00, *P* = 0.031). This suggests that infected and uninfected *Daphnia* in general have distinct sets of bacterial taxa. The Weighted UniFrac distance which weighs differences in ZOTUs by their relative abundance and phylogenetic relatedness was significantly different between *Caullerya-*infected and uninfected *Daphnia* in the guts (Fig. 2B, F = 3.115, *P* = 0.0386), but not the *Daphnia* bodies (Fig. 2D, Table 2). This implies that the distinct bacterial taxa in infected *Daphnia* guts (but not bodies) are also phylogenetically distinct and differentially abundant compared to uninfected guts.

**Table 2.** One-way PERMANOVAs of beta diversity metrics Jaccard Dissimilarity (A) and Weighted Unifrac Distance (B) across *Caullerya*-infected and uninfected *Daphnia* (9999 permutations). Analyses were conducted separately for each tissue type, indicated on the left. %Variation column shows the % variation explained by the model using db-RDA. *P < 0.05

(A)	Jaccard Dissimilarity							%Variation
**gut**		Df	SumsOfSqs	MeanSqs	F.Model	R2	Pr(>F)	
	infection	1	0.59	0.59	3.01	0.188	0.0093*	18.8
	Residuals	13	2.55	0.196		0.812		
	Total	14	3.14			1		
**body**		Df	SumsOfSqs	MeanSqs	F.Model	R2	Pr(>F)	
	infection	1	0.365	0.365	2	0.143	0.031*	14.3
	Residuals	12	2.192	0.183		0.857		
	Total	13	2.556			1		
(B)	**Weighted Unifrac Distance**							**%Variation**
**Gut**		Df	SumsOfSqs	MeanSqs	F.Model	R2	Pr(>F)	
	infection	1	0.848	0.848	3.115	0.193	0.0386*	19.4
	Residuals	13	0.354	0.027		0.803		
	Total	14	0.437			1		
**body**		Df	SumsOfSqs	MeanSqs	F.Model	R2	Pr(>F)	
	infection	1	0.028	0.028	1.694	0.124	0.1536	12.5
	Residuals	12	0.197	0.017		0.876		
	Total	13	0.225			1		

### Indicator taxa

**Figure 3.**
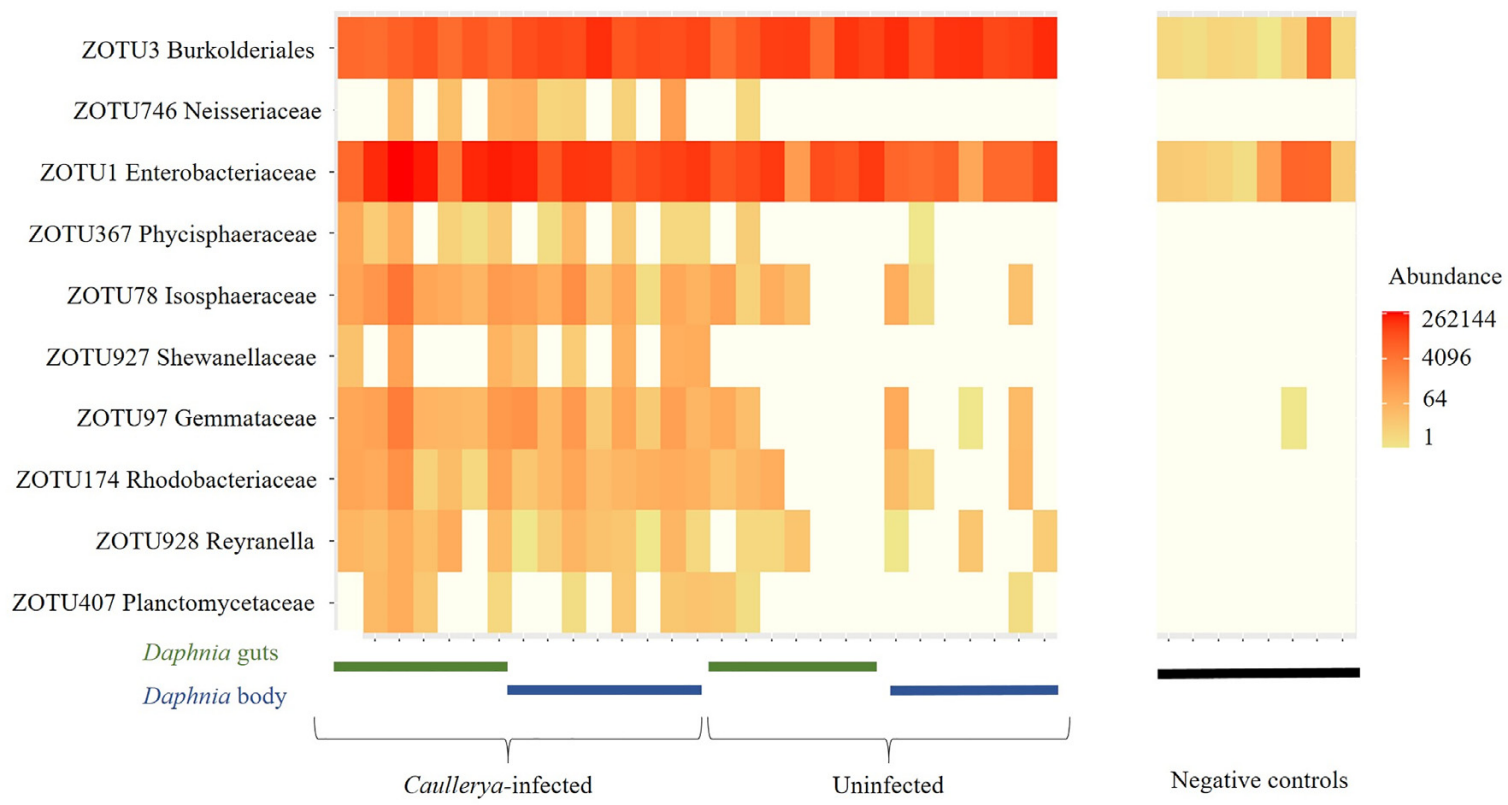
Heatmap of log-transformed ZOTU counts, showing abundance of ZOTUs that indicate presence or absence of *Caullerya* infection in *Daphnia* samples, as identified by Indicspecies. Rows correspond to ZOTUs (with only the family shown) and columns represent infected or uninfected samples (*Daphnia* gut, *Daphnia* body or negative control, as indicated. See methods for details on negative controls.)

Indicator analysis resulted in 10 ZOTUs that were indicative of *Caullerya* infection status, including two ZOTUs that were highly abundant in the dataset. Nine out of the ten discovered indicator ZOTUs were more abundant in *Caullerya-*infected than in uninfected *Daphnia* (Fig. 3, Table S4).

### Dominant bacterial orders

**Figure 4.**
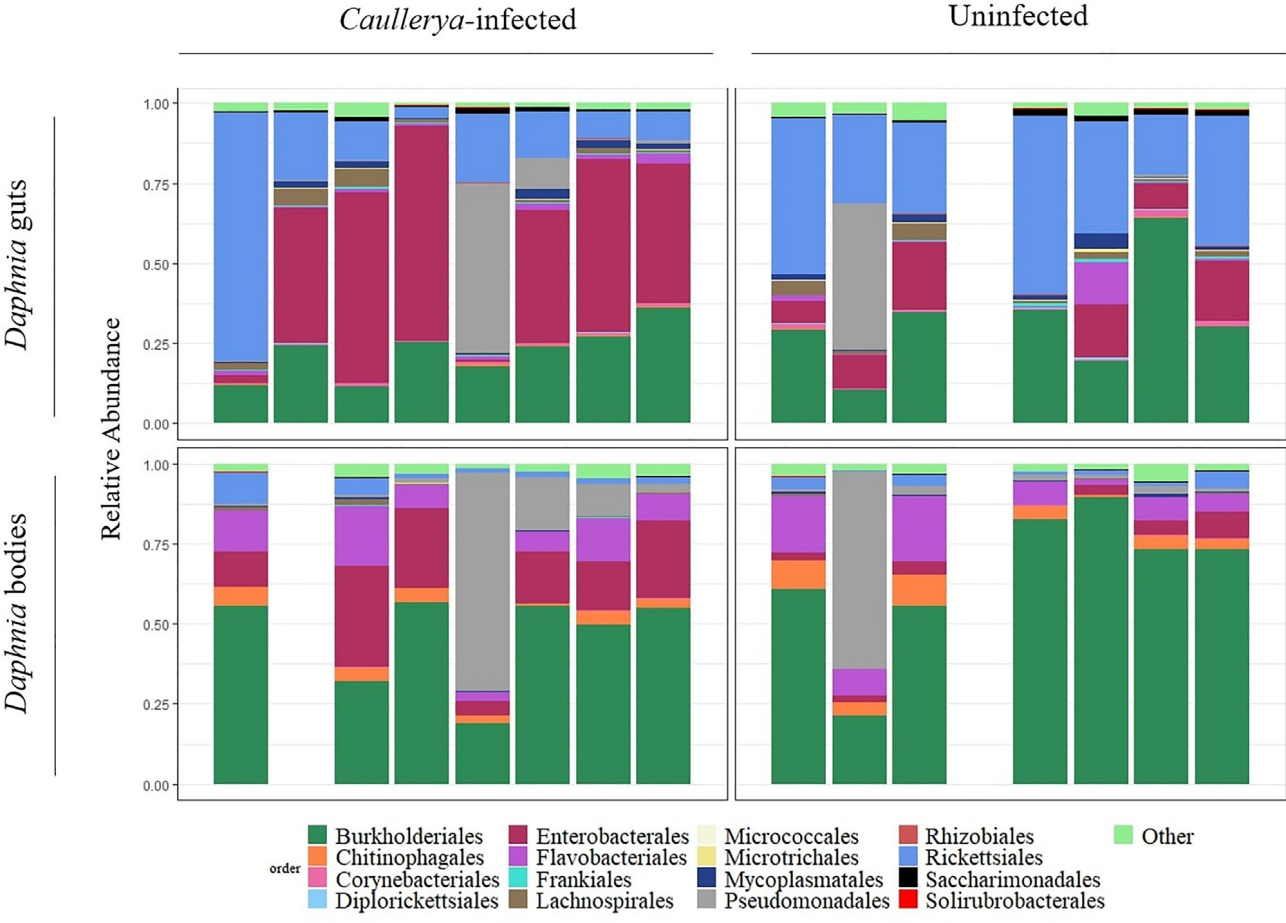
Bacterial communities in *Caullerya*-infected (left panels) and uninfected (right panels) *Daphnia*. *Daphnia* gut communities are shown on the top and body communities at the bottom. Rare taxa, i.e. bacterial orders comprising <1% of the total dataset and not present in every sample were classified as ‘Other’, containing 90 orders. Empty spaces correspond to samples that failed sequencing and hence were not part of analysis.

Gut and body bacterial communities of *Daphnia* sampled from lake Greifensee comprised of 18 dominant orders (Fig. 4). Bodies of infected *Daphnia* had a higher relative abundance of Enterobacteriales (18.21 ± 9.23%, mean ± SD) and Pseudomonadales (14.37 ± 24.47%) compared to bodies of uninfected *Daphnia* (3.54 ± 2.58% and 10.29 ± 22.58%, respectively). Micrococcales were rare within *Daphnia;* but significantly more abundant in uninfected bodies (0.009 ± 0.006%) compared to infected bodies (0.005 ± 0.003%). The above bacterial orders that differed by infection status in *Daphnia* bodies followed a similar (although not significant) trend in *Daphnia* guts. Enterobacteriales constituted 38.9 ± 24.77% of *Caullerya-*infected guts compared to 11.7 ± 7.45% of uninfected guts, while Micrococcales formed 0.027 ± 0.034% of uninfected guts compared to 0.014 ± 0.009% of *Caullerya-*infected guts (Fig. 4). Nevertheless, dominant bacterial orders varied substantially across replicates; two gut-body pairs (one *Caullerya-*infected and one uninfected) were dominated by the order Pseudomonadales, which was typically less abundant in all other samples (see Tables S2 and S3). Moreover, one *Caullerya-*infected gut sample (replicate 1 in top, left panel of Fig. 4) unexpectedly showed a bacterial order composition more like uninfected *Daphnia* guts. We repeated the analysis of differential abundance in dominant bacterial orders after removing this sample and found that the order Rickettsiales was significantly more abundant in uninfected (36.36 ± 12.89%) compared to infected (12.84 ± 6.66%) *Daphnia* guts (W = 3.83, *p*adj *=* 0.01, data not shown).

## Discussion

In this study, we compared the bacterial communities of gut and body tissue between *Caullerya-*infected and uninfected *Daphnia* in the wild. In agreement with our predictions, host gut bacterial communities were significantly less even (as indicated by a lower Shannon Index), indicating an abundance shift of some bacterial taxa. Further, gut bacterial communities of infected and uninfected hosts also differed in their beta diversity indices, confirming our hypothesis that infected hosts have a distinct bacterial community composition compared to uninfected hosts. However, we also hypothesized changes in bacterial community composition would apply only to the host gut but not body tissue, since the investigated infection is gut-specific. For this, we got mixed results: in support of our hypothesis, *Daphnia* gut but not body bacterial communities differed in their Weighted UniFrac Distances. But contrary to our hypothesis, body bacterial communities varied in Jaccard Dissimilarity, suggesting that *Daphnia* body tissue also have distinct bacteria in the presence of a gut infection. Further, infected and uninfected hosts had distinct associated ZOTUs. Overall, these patterns are consistent with changes in host-associated bacterial communities concurrent with infection, as reported in other wild systems such as mallards (Ganz *et al*. 2017) and bats (Wasimuddin *et al*. 2018).

In the present study, the visual presence of parasite spores in the host gut is an indicator of late-stage infection; fully mature spores in the gut are visible 8–12 days post initial exposure to the parasite (Lohr *et al*. 2010). Therefore, during an ongoing epidemic in the field, the hosts classified as uninfected in our study could include individuals with undetected earlier stage infections. This may underlie the substantial variation between replicates among dominant bacterial orders detected in the guts of uninfected *Daphnia*. Nevertheless, we find that the gut bacterial communities of infected hosts with visible parasite spore clusters are distinct in alpha and beta diversity from those of uninfected and/or early stage infected hosts. This suggests that the infection most likely preceded the formation of distinct bacterial communities compared to uninfected hosts.

A previous experiment in *Daphnia galeata* reported a suppression of immune-related gene expression through a downregulation in C-type lectins, which play a role in pathogen cell recognition, 48 h after exposure to *Caullerya* (Lu *et al*. 2018). C-type lectin expression is intricately linked with host microbiota; in the kuruma shrimp *Marsupenaeus japonicus*, C-type lectin expression regulates gut bacterial community composition (Zhang *et al*. 2021). Conversely, intestinal microbiota epigenetically downregulate C-type lectin expression in the mouse host to prevent adherence by a gut pathogen (Woo *et al*. 2019). We speculate that such a downregulation of immune-related genes in the host in the present study may also contribute to increased ZOTU richness associated with the bacterial communities of infected *Daphnia* bodies (Fig. 1), since more bacteria may colonize hosts with a suppressed immune response.

Our results do not eliminate the possibility of reverse directionality, i.e. associated bacteria also playing a role in susceptibility to infection. Epidemics of infectious diseases are often triggered by the host population experiencing an environmental stressor (Gehman *et al*. 2018, LaDeau *et al*. 2015, Lafferty and Holt 2003), which may indirectly impact population health by altering host-associated bacterial communities (Greenspan *et al*. 2020). Outbreaks of *Caullerya* in wild *Daphnia* populations correlate with cyanobacterial blooms, as confirmed with field observations and lab experiments (Tellenbach *et al*. 2016). Cyanobacterial blooms are associated with dramatic changes in the structure of lacustrine pelagic bacterial communities (Tromas *et al*. 2017), whereas freshwater zooplankton such as *Daphnia* undergo considerable shifts in associated bacterial communities driven by changes in environmental bacterial communities in general (Callens *et al*. 2020, Eckert *et al*. 2021) and by exposure to cyanobacteria in particular (Macke *et al*. 2017). At the time of *Daphnia* collection for the present study, the lake had a large bloom of cyanobacteria, particularly of the genus *Gomphosphaeria* (unpublished data). We speculate that a cyanobacterial diet or an altered pelagic bacterial community concurrent with cyanobacterial blooms may have initiated a perturbation in host bacterial communities, predisposing them to infection and the infection then further altered bacterial community structure.

The bacterial community structure of infected and uninfected hosts at the order level also points to physiological stress in the host population in the present study. An overgrowth of the family Enterobacteriaceae is associated with intestinal inflammation and dysbiosis in mammals including mice and humans (Chong *et al*. 2020, Lupp *et al*. 2007, Zeng *et al*. 2017). Though the family Enterobacteriaceae (particularly ZOTU1, Family: Enterobacteriaceae) was more abundant in infected hosts (29.00 ± 21.38%), it was also among the dominant taxa in uninfected hosts (8.00 ± 6.8%). Enterobacteriaceae was described as a core taxon in rotifers and crustaceans (including *Daphnia*) in one study (Eckert *et al*. 2021), but other studies have showed this family was typically rare (Akbar *et al*. 2021, Callens *et al*. 2018, Macke *et al*. 2020) or absent (Freese and Schink 2011) in laboratory-cultured, healthy *Daphnia* guts. Thus, the increased abundance of this family suggests a stressed host population independent of infection, possibly mediated by blooms of cyanobacteria in the lake.

Changes in bacterial community structure, particularly an increased prevalence of opportunistic pathogens, may interact with the primary causative agent of a disease to produce a diseased state in the host (Bernardo-Cravo *et al*. 2020, Egan and Gardiner 2016). In the present study, infected hosts contained 10 differentially abundant ZOTUs compared to uninfected *Daphnia*. Several of these are relatively unknown aquatic microbes; hence, apart from their occasional association with aquatic hosts, little is known about their functional ecology or pathogenic potential (see Table S5). The ZOTUs significantly more abundant in infected hosts contain metabolically diverse species; several of the highlighted bacterial genera or families, e.g. ZOTU927 (Genus: *Shewanella*), ZOTU174 (Family: Rhodobacteraceae) and ZOTU97 and ZOTU78 (Phylum: Planctomycetes) contain both opportunistic pathogens and symbionts with probiotic properties aiding disease resistance in a range of hosts. ZOTU3 (Family: Burkholderiales), which showed an increased abundance in uninfected *Daphnia* is a documented beneficial symbiont of *Daphnia* (Cooper and Cressler 2020, Peerakietkhajorn *et al*. 2016) and is consistently reported as the most abundant host-associated bacterium across *Daphnia* species (Qi *et al*. 2009).

One aspect of host-parasite-bacterial community interaction that remains unaddressed in our study is the role of the host genotype in these interactions. The *Daphnia—Caullerya* host-parasite system is characterized by genetic specificity of infection (Turko *et al*. 2018, Yin *et al*. 2012). Susceptibility to infection is subject to frequency dependent selection between the host and parasite (Wolinska *et al*. 2006), i.e. at a given time point, some host genotypes may be over- or underinfected compared to a random sample of the population (Turko *et al*. 2018). Since we did not genotype the hosts in the present study, it is likely that the pools of infected and uninfected samples comprised of partially different genotypes and/or species of the hybridizing complex. Nevertheless, the differences in bacterial community composition between infected and uninfected samples in the present study is unlikely to be driven by host genetic factors for the following reasons. First, we found that differences by infection status in the present study were primarily in the *Daphnia* gut and not body, even though bacterial communities in both types of *Daphnia* tissue show significant variation by host genotype in the laboratory (Rajarajan *et al*. 2022), suggesting that the observed differences are primarily driven by infection status. Second, the extent of host genotype influence on *Daphnia*-associated bacterial communities is still debated, with conflicting results in laboratory studies (Callens *et al*. 2020, Frankel-Bricker *et al*. 2020, Rajarajan *et al*. 2022, Sullam *et al*. 2018). Bacterial communities also do not show a signal of phylosymbiosis between *Daphnia* species (among other zooplankton) (Eckert *et al*. 2021) or diverge with host genetic distances within the *D. longispina* species complex (Rajarajan *et al*. 2022), suggesting that distinct *Daphnia* species do not have fundamentally different bacterial communities. Further, *Daphnia* genotypes that vary in their bacterial community composition in laboratory settings do not vary similarly when raised in a natural lake environment (Hegg *et al*. 2021). These studies together suggest that host genetic factors alone may not drive the observed differences by infection status in the present study.

However, host genotype and infection status may interact to shape bacterial communities in a host, possibly mediated by host genotype specific immune responses to infection. In sticklebacks, exposure to a parasitic helminth initially causes divergent, host genotype-specific changes in gut bacterial communities that further associate with host genotype-specific immune gene regulation (Hahn *et al*. 2022). Thus, further studies correlating host genotype, infection by a parasite and bacterial community function in the wild would be required to disentangle the complex interaction of various factors that influence host bacterial community structure and its role in host-parasite dynamics. Nevertheless, studies like the present one, which investigate the bacterial communities of wild hosts experiencing multiple environmental stressors, could provide insight into the ecological and evolutionary significance of host-associated microbiota.

## Data availability

Processed 16S sequences are available in DDBJ (DNA Data Bank of Japan) under accession numbers LC686586 through LC687351. Data and R code are available at https://doi.org/10.25678/0005Z6.

## Supplementary Material

fiac097_Supplemental_File
